# Genetics of Suicide

**DOI:** 10.3390/genes16040428

**Published:** 2025-04-03

**Authors:** Mostafa Khalil, Anil Kalyoncu, Alfredo Bellon

**Affiliations:** 1 Brown University, Department of Psychiatry and Human Behavior, Warren Alpert Medical School, Providence, RI 02912, USA; mostafa_khalil@brown.edu; 2 Penn State Hershey Medical Center, Department of Psychiatry and Behavioral Health, Hershey, PA 17033, USA; akalyoncu@pennstatehealth.psu.edu; 3 Penn State Hershey Medical Center, Department of Pharmacology, Hershey, PA 17033, USA

**Keywords:** depression, serotonin, BDNF, inflammation, GABA, cortisol, stress

## Abstract

Over the past two decades, suicide has consistently ranked among the leading causes of death in the United States. While suicide deaths are closely associated with uicidal ideation and attempts, these are not good predictors of future suicide deaths. Establishing who is at risk of suicide remains a challenge that is mostly hampered by the lack of understanding of its pathophysiology. Nonetheless, evidence continues to accumulate suggesting that suicide is driven by a complex and dynamic interaction between environmental factors and genetics. The identification of genes that place people at risk of suicide remains elusive, but data are rapidly evolving. In this narrative review, we describe how Tryptophan hydroxylase (TPH) genes, particularly *TPH1* and *TPH2*, have been associated with suicide in various publications. There is also replicated evidence linking the brain-derived neurotrophic factor gene to suicide, with its most consistent results originating from epigenetic studies. Not surprisingly, many genes involved in the hypothalamic–pituitary–adrenal axis have been connected with suicide, but these data require replication. Finally, among the inflammatory genes studied in suicide, only specific polymorphisms in *TNF-alpha* and *IL-6* may increase susceptibility to suicidal behavior. In conclusion, significant work remains to be performed as inconsistencies undermine the reliability of genetic results in suicide. Potential avenues for future research are proposed.

## 1. Introduction

Over the past two decades, suicide has consistently ranked among the leading causes of death in the United States [1]. In 2022, it was the 11th leading cause of death across all age groups [1]. Notably, suicide was the second leading cause of death for individuals aged 10–14 and 20–34, and the third leading cause for those aged 15–19 [2]. The age-adjusted suicide rate has shown fluctuations over the years. From 2002 to 2018, the rate increased from 10.9 to 14.2 per 100,000 standard population. This was followed by a decline through 2020, reaching 13.5, but then rising again to 14.2 in 2022 [3]. The rise in suicide rates has been observed among both males and females across various age groups. In 2022, the age-adjusted suicide rate for males was 23 per 100,000, while for females, it was 5.9 per 100,000 [4].

Suicide deaths are closely associated with suicidal ideation and attempts, with prior attempts being a predictor of future suicide deaths [5]. However, the World Health Organization estimates that for each suicide death, there are more than 20 suicide attempts [5]. In the United States, the 2022 National Survey on Drug Use and Health reported that 0.6% of adults aged 18 or older attempted suicide in the past year, equating to approximately 1.6 million adults [6]. Additionally, 5.2% of adults experienced serious thoughts of suicide during the same period, underscoring the relationship between suicidal ideation and attempts [6].

The increase in suicide rates in the United States is a critical issue, as suicide is a complex phenomenon involving the interaction of social, psychological, and biological factors. An interesting aspect is the variance in suicide prevalence by age, ethnicity, and residence. Suicide rates are not homogeneous across all groups in the US. For example, males consistently have higher suicide rates compared to females [7]. Non-Hispanic American Indian and Alaska Native individuals and non-Hispanic White individuals have persistently higher rates [7]. In 2022, the highest age-adjusted suicide rate for males was among non-Hispanic American Indian and Alaska Native individuals, at 39.2 per 100,000 [7]. Older adults (65 years and older) also have higher suicide rates. In 2022, individuals aged 85 and older had the highest rates of suicide, at 23.02 per 100,000 [8]. Some of these disparities have changed over time. For instance, in 1999, the age-adjusted suicide rate was 1.4 times higher among rural populations compared to urban populations. By 2018, this difference had increased to 1.8 times, which may reflect societal influences on suicide phenomena, as well as access to mental health care [9].

There is also a significant difference in suicide incidence between states in the US. For example, in 2021, Wyoming had the highest suicide rate, at 32.3 per 100,000 people, while New Jersey had the lowest, at 7.1 per 100,000 [10]. This variation may be due to differences in population characteristics, such as age and ethnicity, urbanization degree, and access to mental health services, as well as differences in suicide reporting systems between states.

Other well-documented circumstances related to suicide include relationship and family conflict, unemployment, low socioeconomic status, social isolation, and incarceration [11,12,13]. Such conditions may also contribute to the variation in suicide rates between states, along with other factors such as the availability of firearms [14,15,16,17]. Additional conditions associated with the risk of suicide include hopelessness [18,19], impulsivity [20,21], and child abuse [22].

While evidence continues to accumulate suggesting that suicide is driven by a complex and dynamic interaction between environmental factors and genetics, the determination of which specific genes place people at risk of suicide remains under intense study. Data are rapidly evolving; therefore, in this narrative review, we present recent information that associates specific genes with suicide (Table 1).

## 2. Methodology

Our objective was to gather recent data about genes associated with suicide and suicidal behavior in an unbiased approach. To achieve this goal, we searched manuscripts by using terms such as “suicide and genetics”, “genes and suicide”, and “methylation and suicide” in PubMed and Google Scholar. Cited references within already selected manuscripts that were relevant to suicide and genetics were also retrieved. Our search was limited to publications in English that studied humans. It is important to note that we did not perform a systematic review of the literature. Instead, our work should be considered a narrative review.

## 3. Results

### 3.1. Family and Twin Studies

#### 3.1.1. Twin Studies

Evidence for a biological predisposition to suicide comes from twin studies, which show that the rates of suicide in monozygotic (MZ) twins are significantly higher than in dizygotic (DZ) twins. A meta-analysis in 2007 reviewed 19 previously published case reports of suicide in twins and five twin register-based studies [23]. The case reports represented 23 twin pairs (20 MZ; 3 DZ), with proband-wise concordance rates found to be 70% for MZ twins and 40% for DZ twins; however, this difference was not statistically significant. In contrast, aggregating the five previously published twin register-based studies, which comprised 492 twin pairs, revealed proband wise concordance rates of 19.5% for MZ twins and 2.3% for DZ twins, a highly significant difference (OR = 6.71, 95% CI = 2.21–20.35; *p* = 3.6 × 10^−6^, Fisher’s exact test). Even after adding the non-significant case report data to the total of twin register studies, the result remained statistically significant, albeit with a slightly lower odds ratio of 5.04 (95% CI = 1.82–13.95; *p* = 4.1 × 10^−8^, Fisher’s exact test). To place these results into perspective, such odds ratios suggest that there is a genetic component that could place individuals at risk of suicide.

#### 3.1.2. Family Studies

In line with twin studies, family studies have shown a genetic predisposition for suicide, which might be partially independent of psychiatric disorders. Numerous family studies in the literature have demonstrated the aggregation of suicide in certain families [24,25,26]. Population-based studies in Denmark and Sweden have found increased rates of suicide in the offspring of suicidal parents compared to the offspring of non-suicidal parents, with no increase in the offspring of parents who died by homicide or accidents [95,96,97].

Psychiatric disorders also aggregate in families and show a strong heritability component. Almost all mental disorders are associated with a higher suicide risk than the general population [98]. It is estimated that 90% of suicide deaths are associated with psychiatric disorders [99], with mood disorders being particularly significant [100]. Does this mean that psychiatric disorders can fully explain the familial risk of suicide? Tsai et al. (2002) found an increased risk of suicide in relatives of bipolar suicide victims compared to relatives of bipolar patients who were not suicidal [101]. Tsuang et al. and Powell et al. (2000) observed higher rates of suicide within families of psychiatric inpatients who completed suicide compared to families of inpatients who were not suicidal, regardless of the inpatient’s psychiatric diagnosis [24,102]. A family study of the Amish community from 1880 to 1980 found 26 reported suicides aggregated within four families, which also had a high incidence of mood disorders. The study also identified other families affected by multiple mood disorders but with no history of suicidal behavior [103]. This highlights that while suicide frequently co-occurs with psychiatric disorders, it may have a partially independent biological pathway.

#### 3.1.3. Population-Based Twin Studies

The concept of the partial independence of suicide from psychiatric disorders is supported by population-based twin epidemiological studies. These studies provide a unique quantification of the interplay between genetic and environmental risk factors for suicidal behavior in twins. Two large studies in Australia and the USA found that the heritability of suicidal behavior (suicidal thoughts, plans, attempts) is 45–48%, with 44% of the variation attributable to non-shared environmental factors and 8% to shared environmental factors [27,28]. Unfortunately, these studies did not control for the presence of psychiatric disorders. However, Fu et al. [29], after controlling for the inheritance of psychiatric illnesses in twin males who served in the military, found slightly lower but still significant rates of suicidal heritability. In this study, the contributions to the variation in lifetime suicidal ideation were 36% additive genetic, 64% non-shared environmental, and 0% shared environmental. For lifetime suicide attempts, the contributions were 17%, 64%, and 19%, respectively. Such data provide evidence that, at least in men, there are genetic components specific to both suicidal ideation and suicide attempts that are distinct from the genetic factors of common psychiatric disorders. Converging evidence from twin and family studies indicates that suicide might involve a disturbed underlying biological pathway that co-occurs mostly with psychiatric disorders but is distinct in its genetic and mechanistic pathway.

Among mental health providers, it is observed that only a small proportion of patients with mood or psychotic disorders attempt suicide [104,105], suggesting that while mood disorders may be one risk factor for suicide, additional genetic factors likely play a role. Converging evidence from twin and family studies indicates that suicide may involve a disturbed underlying biological pathway that co-occurs mostly with psychiatric disorders but is distinct in its genetic and mechanistic pathway. This was supported by the recently performed genomic-wide analysis study of 29,782 suicide attempters that found two loci associated with suicide; one of them was an intergenic area on chromosome 7 that remained associated with suicide even after correction for psychiatric disorders. This area is associated with risk taking, insomnia, and smoking [106]. 

### 3.2. Serotonin System

#### 3.2.1. Tryptophan Hydroxylase

Tryptophan hydroxylase 1 (TPH1) is predominantly expressed in peripheral tissues and not in the central nervous system (CNS). Numerous studies have reported an association between certain *TPH1* polymorphisms and suicidal behavior. For example, a study by Brezo et al. followed 1255 subjects for over 22 years and concluded that a *TPH1* variant (rs10488683) is associated with suicide attempts [30]. This association is independent of gender or psychiatric disorders and is not related to childhood abuse. The association between *TPH1* polymorphisms and suicide was also found to be significant in three meta-analyses that aggregated studies conducted on European and Asian samples [31,32,33]. However, the meta-analysis by Saetre et al. selected only studies comparing allele frequencies in suicidal and non-suicidal psychiatric disorder patients and found no significant difference between the two groups [107]. This suggests that *TPH1* might be associated with an increased risk of psychiatric disorders in general and not specifically with suicide.

Tryptophan hydroxylase 2 (TPH2) is particularly relevant because it is mainly expressed in neurons. Although 8 studies have found an association between *TPH2* polymorphisms and suicide [34,35,36,37,38,39,40,41], a meta-analysis examining 37 studies comparing suicide completers and suicide attempters versus healthy controls did not find an association between (G-703 T, A-473 T, and G19918A) variants of *TPH2* and suicidal behavior [42]. In this meta-analysis, the authors did not perform a separate analysis for suicidal ideation, suicide attempt, or completed suicide, instead, all were included as suicidal behaviors. Another potential explanation for these contrasting results is that several studies including different SNP variants were not included in the meta-analysis [36,37,38,39,40,41].

Another explanation for such discrepancy may be due to differences in methodology. While the meta-analysis included healthy controls, other studies [33,37,39] showing a link between *TPH2* and suicide were compared between patients with depression, while one study involved only patients with bipolar disorder [41]. Additionally, the meta-analysis concentrated on G-703 T, A-473 T, and G19918A variants; meanwhile, other studies turned their attention to different variants, such as rs4448731, rs4641527, rs1386494, rs7305115, and rs1386483.

#### 3.2.2. Serotonin Receptor 1A

Several studies have investigated the association between serotonin receptor 1A (*5-HT1A*) variants and suicidal behavior. Wasserman et al. found over-transmission of the G allele of the C(-1019)G polymorphism (rs6295) and stressful life events prior to suicide attempts, using a transmission disequilibrium test in a sample of 272 families, primarily of Ukrainian and Russian descent [43]. But no association was evident between actual suicide attempts and G allele over-transmission. The same polymorphism was shown to be related to suicide in an Iranian population among 191 suicide victims compared to 218 healthy controls [44]. The same study also showed a correlation between the GG genotype and higher stressful life events among the suicide group. Additionally, Lemonde et al. reported that the G(-1019) allele was twice as enriched in depressed patients compared to non-suicidal individuals and was four times more prevalent in suicide cases [45]. Similar results were obtained in a different study which found a correlation between the same polymorphism and suicide attempts compared to healthy controls [46]. On the other hand, data coming from a Slovenian population comparing 323 suicide victims with 190 controls evidenced no correlation between the C(-1019)G polymorphism and suicide [47]. Another study on individuals with alcohol dependence and suicide attempts also reported no significant association with the rs6295 polymorphism [48].

One meta-analysis including four studies, with one of them being performed by Angles et al., did not show any correlation between C(-1019)G polymorphism and suicidal behavior [49]. However, this meta-analysis included both suicide attempts and completed suicide in the same sample, while no separate analysis for suicide attempts and completed suicide was performed. An updated meta-analysis with 13 case–control studies involving 2817 suicidal behavior patients and 2563 healthy individuals reported a correlation between the C(-1019)G polymorphism and increased suicidal behavior.

The inconsistencies in these findings may stem from population-specific genetic differences, as the association between the C(-1019)G polymorphism and suicide was observed in an Iranian population but not in Slovenian, Russian, or Ukrainian cohorts. Environmental factors, such as the higher prevalence of stressful life events among GG genotype carriers in the Iranian and Ukrainian populations, may have influenced the observed genetic effects. Another potential confounder is the analysis of both suicide attempts and completed suicide as one entity without distinguishing between them, potentially masking genotype-specific effects on different suicidal behaviors.

#### 3.2.3. Serotonin Receptor 1B

Zouk et al. found a significant association between a *5-HTR1B* A-161T variant and impulsive aggressive behavior, but they failed to find any correlation between completed suicide and T-261G, A-161T, C129T, G861C, and A1180G variants [50]. Murphy et al. found a correlation between G861C polymorphism and suicide attempt in a group of 159 patients with Axis I psychiatric diagnosis [51]. However, many other studies found no association between G861C polymorphism and suicidal behavior, including a meta-analysis by Kia-Keating et al. that combined evidence from 789 cases and 1247 controls [52].

#### 3.2.4. Serotonin Receptor 2A

There have been multiple studies investigating the relationship between the *5HT2A* gene polymorphism and suicidal behavior. T102C polymorphism was not associated with suicide attempts among Jewish Ashkenazi adolescents [53]. Another study did not find a link between suicidal ideation and T102C polymorphism in psychotic illnesses [54], whereas a different −1438A/G polymorphism was unrelated to suicide attempts in patients with depression [55]. Similarly, a longitudinal study investigating the relationship between four different *5HT2* variants, rs7997012, rs6313, rs643627, and rs17288723, and suicide risk after at least four weeks of antidepressant treatment did not show any significant correlation between these SNPs and suicide risk or suicide history [56]. Interestingly, a study by Fanous et al. suggested that the −1438G/G genotype was more common in impulsive suicide attempts, while the A/A variant was more common in planned attempts. However, this was not significant after Bonferroni correction [57]. Although Yoon et al. reported an association between the C102C genotype of *HTR2A* and suicide attempts in women with alcohol-use disorder, the association was not significant among men [48]. A meta-analysis by Saiz et al. that aggregated 25 studies did not find any significant association between the T102C variant and suicide. Nonetheless, the same analysis of seven studies examining the A1438G variant reported a significant association with suicidal behavior [58]. A more recent meta-analysis by Höfer et al. in 2016 encountered comparable results, as the authors did not find any association between the 102T/C genotype and suicide attempts [59]. Only the C/C genotype of the *5-HT2A* receptor 102T/C was found to be related to suicide attempts in schizophrenia.

#### 3.2.5. Serotonin Transporter

Several candidate-gene studies have examined the association between suicide and the long vs. short allele of the serotonin transporter gene, with mixed results. Some studies reported a significant association [60,61,62], while others did not [63,64]. Biologically, the long allele is 2–3 times more efficient in transcription of the transporter than the short allele. Three meta-analyses were conducted to verify these results. Two of them [33,108] found a significant association between the short allele and suicidal behavior. However, an initial analysis by Lin and Tsai [109] did not find an association between the short allele and suicide. Interestingly, when a separate analysis compared only patients with the same psychiatric disorder, the frequency of the short allele was more significant among patients who attempted suicide.

### 3.3. Brain-Derived Neurotrophic Factor (BDNF)

Numerous candidate-gene studies have shown associations between the Val66Met (rs6265) polymorphism in the *BDNF* gene and suicide. Interestingly, this association has been found in the context of various psychiatric diagnoses, including schizophrenia [65], bipolar disorder [66,67], depression [68,69], and alcohol-use disorder [70], among several ethnic groups (Caucasian, Japanese, Chinese, and Slovenian). These findings suggest that certain genotypes in the *BDNF* region might serve as mediators for the risk of suicide among different psychiatric disorders.

Replicating results in genomic studies remains a challenge. For instance, Zarrilli conducted a postmortem analysis on DNA extracted from the Wernicke-area samples of 262 suicide subjects and 250 non-suicide control subjects [110]. This study encountered no association between *BDNF* Val66Met and suicide, which contradicted an earlier study by the same team which had found an association between Val66Met and suicidal behavior. The authors speculated that they could not replicate the association because the second study involved suicide completers instead of suicide attempters, suggesting different molecular pathways for these behaviors. However, another plausible explanation is that the second study did not control for psychiatric disorders, unlike the first study, which included patients with major depressive disorder (MDD). In a study of multiplex bipolar families, Sears et al. [71] found an association between five SNPs of *BDNF* and suicide attempt, but the association was not significant after the corrections. Instead, they identified an association between a polymorphism in the cholecystokinin B (*CCKB*) gene and suicide, controlling for bipolar disorder to validate that the association was with suicide, not bipolar disorder.

Zai et al. [72] conducted the first meta-analysis of the functional *BDNF* marker Val66Met (rs6265, 196G > A) in suicidal behavior, using data from 12 studies in mainly Caucasian populations (total *n* = 3352 subjects, of whom 1202 had a history of suicide). This meta-analysis revealed a trend indicating that the Met allele and Met-carrying genotypes conferred a risk of suicide (*p* = 0.032; OR Met = 1.16, 95% CI 1.01–1.32). These statistical results are not particularly strong considering the odds ratio is barely above 1.

A meta-analysis by Ratta-Apha in a Japanese population found an association between Val66Met and suicide attempts but not suicide completion [73], highlighting a potential distinction in pathways between these behaviors. Similarly, Clayden’s meta-analysis found an association between suicide attempts and ideation with serotonergic receptors and *TPH* but not with *BDNF* polymorphism [33]. This analysis included only seven of the twelve studies from Zai et al. [72] due to stringent inclusion criteria. A common theme across these three meta-analyses is the distinction between suicide completion and attempts: two found associations between *BDNF* and suicidal behavior but not completion, while the third found no association.

Findings from epigenetic studies appear more consistent. Hypermethylation of the *BDNF* promoter region is associated with reduced BDNF production [74]. Keller et al. [75] found significant hypermethylation of the *BDNF* gene in 18 suicidal patients compared to 18 controls in the Wernicke area in a postmortem study. This finding was replicated using three independent quantitative methylation techniques in postmortem samples of 44 suicide completers and 33 controls of Caucasian ethnicity [76].

In three consecutive Korean studies, Kang et al. [111] associated higher scores on Beck’s Scale for Suicidal Ideation with hypermethylation of the *BDNF* promoter region in a sample of patients with depression. This association remained significant after controlling for confounders such as depression severity and functioning. This finding was replicated in two consecutive studies in cohorts of geriatric patients with MDD [77] and a group of females who had undergone breast cancer surgery and had MDD [78].

Notably, a genome-wide epigenetic study found the most significant methylation differences in genes involved in cellular and neuronal plasticity in a sample of suicide completers who had experienced severe childhood abuse. Although the robustness of the result cannot be determined, it raises the question of whether the epigenetic signal is related to suicide risk or early life adversity [112].

### 3.4. Hypothalamic–Pituitary–Adrenal (HPA) Axis

The HPA axis is the main stress regulatory system in the body [84]. Disturbances in the HPA axis have been observed in depressed patients, increasing their risk of suicide. Notably, a meta-analysis of prospective studies using the Dexamethasone Suppression Test (DST) found that non-suppression of cortisol is associated with completed suicide [113]. In another study, DST non-suppressors were estimated to have a fourfold increase in suicide risk [114]. In a 15-year follow-up study of seventy-eight patients with depressive illness, those with abnormal DST results had a 26.8% risk of suicide based on survival analysis, compared to only 2.9% among those with normal DST results [115]. It is important to note that many biological correlates of HPA axis disturbances appear to be linked to childhood trauma or early life adversity.

The heritability of HPA system reactivity ranges from 56% to over 97% [116]. Individual variations in HPA reactivity to stress are influenced by genetic polymorphisms in the GABA-A alpha 6 receptor gene, and mineralocorticoid and glucocorticoid receptor genes [79,80].

#### 3.4.1. Gamma-Aminobutyric Acid (GABA)

GABA, the main inhibitory neurotransmitter in the mammalian central nervous system (CNS), inhibits the HPA axis through its actions on GABA receptors expressed by corticotropin-releasing hormone (CRH) neurons within the paraventricular nucleus (PVN) of the hypothalamus [82]. Additionally, GABA inhibits the locus coeruleus–norepinephrine system, which is a central sympathetic system [83]. A study found that indicators of HPA functioning, such as adrenocorticotropin (ACTH), cortisol, diastolic blood pressure, and mean arterial blood pressure, varied significantly among healthy subjects with different variants of the GABAAα6 receptor subunit gene [80]. Subjects homozygous for the C allele exhibited significantly lower HPA reactivity to social stress and were less extroverted on personality tests [80]. The relationship between GABRA6 and the HPA axis is thought to be mediated by GABA activity on CRH neurons in the paraventricular nucleus of the hypothalamus [82,83].

#### 3.4.2. Corticotropin-Releasing Factor-Binding Protein (CRHBP)

Polymorphisms in the *CRHBP* gene have been found to increase the risk of suicidal behavior, particularly in association with childhood trauma [117]. Glucocorticoid receptors play a crucial role in the feedback inhibition of the HPA system. In a genetic study on the Japanese population, a haplotype of *FKBP5* (FK506 binding protein, which interacts with glucocorticoid receptors) was correlated with suicide [81].

#### 3.4.3. Spindle and Kinetochore-Associated Protein 2 (SKA2)

Methylation and polymorphisms of the *SKA2* gene have been found to correlate with suicide in postmortem brains of suicide subjects; these findings were also replicated in the blood of suicide patients [85,86]. The association of *SKA2* with suicide appears specific across different psychiatric disorders [118]. SKA2 likely mediates suicide risk through its interaction with the glucocorticoid receptor (GR), which is involved in the feedback inhibition of the HPA axis [84,119].

#### 3.4.4. *NR3C1*

Hypermethylation of CpG sites in the *NR3C1* gene, which codes for the glucocorticoid receptor in the hippocampus, including GR1F, was observed in the suicide completers with a history of child abuse compared to the non-abused suicide completers and control groups [87]. Additionally, Steiger et al. compared female patients with bulimia nervosa (BN) to normal controls, examining whether childhood abuse, suicidality, and borderline personality disorder among the eating-disorder group would modify the risk. They found significantly higher methylation of *NR3C1* at specific exon 1C sites in BN patients with comorbid borderline personality disorder or suicidality compared to normal controls [120]. Moreover, decreased expression of the GR variant GR1F (the product of *NR3C1* gene) was found in the hippocampus of suicide completers with a history of child abuse (SCAs) compared to non-abused suicide victims (SCNAs) and controls [87]. This decreased expression likely results in reduced negative feedback on HPA reactivity.

#### 3.4.5. *CRH1*

Lastly, Uhart et al. found no significant difference in *NR3C1* methylation between MDD patients with and without suicidal ideation [80]. However, significant differences in the methylation of the corticotropin-releasing hormone receptor 1 gene (*CRH1*) were observed between the two groups.

### 3.5. Second Messengers

Alterations in markers of second messenger function represent another potential biological endophenotype for suicide. Several markers have been shown to be altered in teenagers and adults who died by suicide, including glycogen synthase kinase-3β (GSK-3β), a key component of the Wnt signaling pathway [121]. Additionally, protein and messenger RNA (mRNA) expression levels of protein kinase C (PKC) isozymes, specifically PKCα, PKCβ, and PKCγ, have been observed to change [88]. Transcription factors, such as cyclic adenosine monophosphate (cAMP) response element-binding (CREB) and brain-derived neurotrophic factor (BDNF), have also been implicated [121]. Lower levels of tyrosine receptor kinase B (TrkB) mRNA and protein have been found in the prefrontal cortex and hippocampus of individuals who died by suicide [122]. However, whether these alterations meet the endophenotype criteria remains to be determined, as data on their heritability, trait status, co-segregation in families, or frequency in non-affected relatives are not yet available.

Focusing on specific pathways, two have been reported to show abnormalities in psychiatric disorders: the phosphatidylinositol (PI) pathway and the adenylate cyclase pathway. Studies, mainly postmortem, have found that two enzymes, PKC (Protein Kinase C) and PKA (Protein Kinase A), exhibit decreased activity and expression in suicide patients compared to controls [88,89]. However, interpreting these findings is challenging because the associations could be with psychiatric disorder or other potential variables.

Pandey et al. found that the Bmax of [3H] PDBu binding and PKC activity were significantly decreased in both membrane and cytosol fractions obtained from the prefrontal cortex (PFC) of teenage suicide victims compared to controls [88]. They also observed that protein and mRNA expression levels of the conventional family of PKC were significantly decreased in the membrane and cytosol fractions of the PFC and hippocampus of teenage suicide victims compared to control subjects.

Regarding PKA, Pandey et al. [89] assessed cAMP binding to PKA, PKA activity, and protein and mRNA expression of different PKA subunits in cytosol and membrane fractions from the PFC, hippocampus, and nucleus accumbens (NA) of postmortem brains from teenage suicide victims and non-psychiatric control subjects. They found that PKA activity was significantly decreased in the PFC but not in the hippocampus of teenage suicide victims compared to controls. Interestingly, the protein and mRNA expression of two PKA subunits, PKA RIα and PKA RIβ, were decreased in the PFC of teenage suicide victims, while other subunits were not. Furthermore, the expression profile of some PKC subunits differed between suicide victims and controls, with variations observed between adults and adolescents.

### 3.6. Inflammation

In mammals, infections and tissue damage can elicit fever, muscle aches, reduced psychomotor activity, sleepiness, decreased interest in social interactions, loss of appetite, anxiety, and neurocognitive impairment. These symptoms significantly overlap with those of depression [123]. Tissue damage and infections induce such symptoms through the release of pro-inflammatory cytokines. Not surprisingly, numerous studies have demonstrated that pro-inflammatory cytokines are elevated in both depression and sickness behavior [124]. These overlaps raise the question of how inflammation affects suicide and whether this could be related to genetic risk factors.

Several meta-analyses reported correlations between inflammatory markers and suicide and suicide attempts [90,125,126]. Black and Miller (2015) conducted a meta-analysis that reported elevated levels of cytokines, particularly interleukin-1β (IL-1β) and interleukin-6 (IL-6), in the blood, cerebrospinal fluid (CSF), and postmortem brain tissue of individuals with suicidality [90]. This includes those with suicidal ideation, suicide attempts, and completed suicides. The most robust associations were observed in completed suicides, highlighting the role of inflammatory processes in the pathophysiology of suicidal behavior. Additionally, IL-8, an anti-inflammatory cytokine, was significantly decreased in suicidal patients compared to the control group in the same meta-analysis [90]. A different meta-analysis published the same year found a medium effect size of low IL-2 plasma levels in suicidal patients compared to both the healthy control group and non-suicidal control groups. In addition, lower IL-4 and higher transforming growth factor (TGF) beta plasma levels were detected in suicidal patients compared to healthy individuals [125]. A meta-analysis with cerebrospinal fluid (CSF) and positron emission tomography (PET) findings on both in vivo and postmortem subjects detected elevated IL-6 in CSF among suicide attempters regardless of their psychiatric diagnosis [126]. The most consistent findings among those studies appear to be elevated IL-6.

#### 3.6.1. Tumor Necrosis Factor (TNF) Alpha

In one of the very first studies related to the immunogenetics of suicide, no association was found in the *TNF alpha* (−308A/G) gene polymorphisms when suicide attempters were compared with controls [127]. Another study from Iran with 154 patients and 160 healthy individuals reported an association between *TNF-alpha* −308G/G with suicidal behavior, but only in men [128]. In a Brazilian sample involving several psychiatric disorders, the −308A/G genotype was significantly associated with suicide attempts, and the −308G allele appeared to be a risk factor for women [91]. In another study with major depressive-disorder patients, *TNF-alpha*−308G/A polymorphism was detected in suicide attempts compared to non-attempters [92].

Regarding epigenetic studies, TNF-alpha expression was higher in the dorsolateral prefrontal cortex (dlPFC) of 12 subjects who had completed suicide and had a history of MDD compared to 12 MDD individuals [129]. The study demonstrated that miR-19a-3p regulates TNF-α expression in vitro and reported elevated levels of miR-19a-3p in the dlPFC of individuals who died by suicide, suggesting its role in the dysregulation of inflammatory pathways linked to suicidal behavior. On the other hand, another study reported lower levels of miR-19a-3p in the CSF of males who died by suicide through hanging compared to those who died from cardiac arrest [94].

#### 3.6.2. IL-6

IL-6 is another pro-inflammatory cytokine that has been shown to be associated with suicide attempts in molecular studies [90,126]. However, genetic reports regarding IL-6 are limited. One study from Iran showed a significant association (OR (95% CI) =1.33 (1.04–1.71) between the rs1800795 C allele and suicide completers compared to suicide attempters. It is important to note that such an odds ratio barely above one indicates that the clinical significance of these data is limited. This allele was also related to the lethality of the suicide attempt in a regressive model used in the same study [93]. But there was no link between the rs2069845 variant of *IL-6* and suicidality.

#### 3.6.3. *IL-8*

Two studies have examined the relationship between the *IL-8* polymorphism and suicide. In one study, the T allele of the rs4073 (−251) polymorphism was observed exclusively in female suicide attempters [130]. In contrast, the other study identified the same T-allele polymorphism across the entire study population, regardless of gender [131].

#### 3.6.4. IL-1 Beta

One study that investigated *IL-1B* −511 C/T polymorphism initially showed a correlation between suicidal ideation in acute coronary-syndrome patients. However, the association was not significant after the Bonferroni correction [132].

### 3.7. Genome-Wide Association Studies

Genome-wide association studies (GWASs) represent a significant advancement in genetic research, enabling the identification of common genetic variants associated with specific traits or disorders by scanning the entire genome in an atheoretical manner. A key advantage of GWASs is their unbiased approach, as they do not rely on prior hypotheses about specific genes. However, GWASs face several challenges: they are prone to false positives due to the large number of statistical tests performed, necessitating stringent significance thresholds (e.g., genome-wide significance, *p* < 5 × 10^−8^) to reduce Type I errors [133,134]. Moreover, GWASs require large sample sizes to detect variants with small effect sizes [135]. Importantly, the identified genetic variants are typically associated with the trait rather than being causative, highlighting the need for functional validation and integrative approaches, such as Mendelian randomization and post-GWAS functional studies, to infer causality [133,134,135].

Several GWAS studies have examined genetic loci correlation with suicide. The risk is overlaps with psychiatric disorders; for example, a large study conducted by Li et al. compared the genome of 3765 cases with suicide deaths to 6572 controls and found a significant loci in neuroligin1 (*NLGN1*) to be significantly associated with suicide death even after conditioning on suicide attempts and on major depressive disorder [136]. The neurexin–neuroligin pathway is critical for excitatory and inhibitory synaptic development across the brain; neurexins (NRXNs) are presynaptic adhesion molecules that bind to NLGN proteins on the postsynaptic membrane. This interaction is fundamental for synaptic structure and allows the interaction of neurotransmitter with its receptors [137]. The NRXN/NLGN pathway has been associated with autism and schizophrenia [138,139,140,141,142,143].

A large GWAS study which compared more than 6500 suicide attempters who had major depressive disorder, bipolar disorder, and schizophrenia to a cohort of controls who had the same diagnosis but without suicide attempt found three loci associated with attempting suicide [144]. One loci was with MDD, one was with bipolar disorder, and the third was mood disorders in general, highlighting that suicide risk was mediated through psychiatric disorders; however, the associations were not replicated in an independent sample [144].

A meta-analysis led by the same author [106] incorporated 29,000 samples of individuals that attempted suicide and compared their genetic variants against more than 500,000 controls. Such a large cohort helped in detecting two loci that were significantly associated with suicide, one in the major histocompatibility complex and another intergenic locus on chromosome 7. The intergenic locus on chromosome 7 remained associated with suicide even after controlling for psychiatric disorders, and of note, this result was replicated in an independent cohort [106]. Replication of findings from genetic studies has always been a limitation, hence the importance of these data. The variant on chromosome 7 is not known to be linked to any specific gene, and this risk allele has been associated before with risk-taking behavior, smoking, and insomnia [145,146,147].

### 3.8. Machine Learning

GWASs and the sequencing of the human genome have held great promise in unraveling the physiological and pathological underpinnings of various biological processes [135]. However, translating GWAS findings into observable clinical traits remains challenging. As discussed earlier, common genetic variants are often associated with multiple traits, necessitating the use of polygenic risk scores to establish meaningful connections to phenotypes such as suicidal behavior [133].

A more recent approach that addresses these challenges is machine learning (ML), which offers significant advantages over traditional statistical methods. Unlike conventional techniques that classify risk factors, ML has four key benefits [148]: according to [133], it autonomously determines the most efficient algorithm for a given task; according to [134], it can handle complex interactions among risk factors more effectively; according to [135], it prioritizes clinical significance over mere statistical associations; and according to [136], it processes high-dimensional data that are difficult to examine with traditional methods, enabling nuanced pattern detection. For instance, Walsh et al. (2017) developed a machine-learning model using electronic health records to predict future suicide attempts [148]. The study analyzed 3250 patients with suicide attempts and 1917 patients with non-suicidal self-injury, constructing a predictive model for suicide risk over periods ranging from one week to two years. Their model outperformed traditional logistic regression, achieving an area under the curve of 0.84 and a precision of 0.79. These statistical results are promising, but the model’s potential clinical significance still has room for improvement.

While ML approaches are promising tools with high predictive accuracy and the ability to process complex data, they are not without limitations. A primary concern is the generalizability of findings, as models trained on specific populations with unique data features may not translate effectively to diverse settings or populations [149].

## 4. Discussion

The exploration of the genetic and biological underpinnings of suicide reveals a complex interplay of factors that contribute to suicidal behavior. Evidence from twin and family studies underscores the significant heritability of suicidal tendencies, with monozygotic twins displaying higher concordance rates for suicide compared to dizygotic twins [23]. This suggests a genetic predisposition, which may be partially independent of psychiatric disorders. Family studies further support the genetic basis of suicide, highlighting the aggregation of suicide within certain families [24,25,26]. Population-based twin studies also emphasize the role of genetic and non-shared environmental factors in suicidal behavior [27,28,29].

Among neurotransmitters’ genes, those related to serotonin appear as promising candidates. Tryptophan hydroxylase (*TPH*) genes, particularly *TPH1* and *TPH2*, have been implicated in suicidal behavior [30]. Variants in these genes have shown associations with suicide in various studies [31,32,33]; however, meta-analyses present mixed results [107], indicating the need for further investigation. Similarly, serotonin receptors, such as *5-HT1A*, *5-HT1B*, and *5-HT2A*, and the serotonin transporter gene (*HTTLPR*) have been studied for their potential links to suicide, with varying outcomes across different populations and study designs [43,44,45,50,51,53,54,55,56,60,61,62]. These findings highlight the complexity of genetic influences on suicide, suggesting that specific genetic variants may interact with environmental and psychological factors to influence suicidal behavior.

The brain-derived neurotrophic factor (*BDNF*) gene, specifically the Val66Met polymorphism, has been extensively studied for its association with suicide [65,66,67,68,69,70]. While some studies and meta-analyses have found significant associations [72], others have not [71], reflecting the challenges in replicating genomic findings. Epigenetic research offers more consistent evidence, with hypermethylation of the *BDNF* promoter region being linked to reduced BDNF production and increased suicide risk [74,75,76,77,78,111]. This suggests that both genetic and epigenetic factors may contribute to the biological pathways underlying suicidal behavior.

The hypothalamic–pituitary–adrenal (HPA) axis, a key stress regulatory system, has also been implicated in suicide. Disturbances in the HPA axis, often linked to childhood trauma or early life adversity, have been observed in depressed patients, increasing their suicide risk [113]. Genetic polymorphisms in glucocorticoid and mineralocorticoid receptor genes influence HPA reactivity to stress, further connecting biological stress responses to suicidal behavior [79].

Another promising research avenue in suicide is second messengers, such as GSK-3β, PKC, and PKA, since they have been found to be altered in individuals who died by suicide [88,89,121]. These alterations in signaling pathways may represent potential biological endophenotypes for suicide, though their heritability and trait status require further exploration.

Inflammation plays a significant role in the overlap between sickness behavior and depression, as both are associated with elevated levels of pro-inflammatory cytokines, such as IL-1B, IL-6, and TNF-alpha. Not surprisingly, numerous studies have demonstrated correlations between these cytokines and suicidal behavior. Meta-analyses revealed that elevated cytokine levels are present in blood, brain tissue, and cerebrospinal fluid of suicidal patients, with IL-6 showing the most consistent association [90,125,126]. Genetic studies further indicate that specific polymorphisms in cytokine genes, such as *TNF-alpha* and *IL-6*, may increase susceptibility to suicidal behavior [90,91,92,93,126,127,128]. Additionally, epigenetic modifications, including changes in miRNA expression, may influence cytokine levels and contribute to suicide risk [94,129].

While the goal of this manuscript is to present genes that have been associated with suicide and suicidal behavior, we do not intend to overemphasize the role of genetics. The complex, multifactorial nature of suicide risk cannot be reduced to an overly deterministic view of genetic influences. There are many factors that influence suicide and suicide risk; among them are early life adversity, personality traits, and cognitive deficits, which have been thoroughly described by Turecki et al. [150]. Other environmental influences that tend to receive less attention but are also relevant consist of political, social, cultural, and economic factors. A comprehensive review on the role of these contributors to suicide was recently undertaken by Professor Stack [151].

## 5. Conclusions

Suicide is a multifaceted phenomenon influenced by a complex interplay of genetic, epigenetic, and environmental factors. Such complexity is likely undermining the reliability of results in suicide research, leading to a continued state of uncertainty in the field. More rigorous research integrating polygenic risk scores, leveraging machine learning for predictive modeling, and conducting large-scale multi-ethnic studies is likely to render more generalizable and impactful results. Moreover, genetic results could be strengthened with a more physiological perspective if assessments of brain organoids and other cellular approaches that originate from patients’ cells [152] and therefore carry the genetic load of suicide are conducted in parallel to large-scale genetic studies.

## Figures and Tables

**Table 1:** Genetic and epigenetic studies in suicide.

Topic	Genetic Findings	Epigenetic Findings
Twin-, Family-, and Population-Based Studies	Higher concordance rates for suicide in monozygotic twins compared to dizygotic twins. Proband-wise concordance rates were 19.5% for MZ twins and 2.3% for DZ twins, a highly significant difference [23]. Family studies show a genetic predisposition for suicide, independent of psychiatric disorders, with higher rates of suicide in the offspring of suicidal parents [24,25,26]. Population-based studies: The heritability of suicidal behavior is estimated at 45–48%, with significant contributions from non-shared and shared environmental factors. Genetic factors contribute to suicidal ideation and attempts, distinct from psychiatric disorders [27,28,29].	N/A
Serotonin system	Tryptophan hydroxylase: Association between *TPH1* polymorphism rs10488683 and suicidal behavior, significant in meta-analyses [30,31,32,33]. *TPH2* polymorphisms have shown mixed results [31,32,34,35,36,37,38,39,40,41,42]. Serotonin receptors: Variable associations with suicide. Some studies show significant links with *5-HT1A, 5-HT1B, and 5-HT2A*, while others do not [43,44,45,46,47,48,49,50,51,52,53,54,55,56,57,58,59]. Serotonin transporter: Mixed results on the association between long vs. short alleles of *5-HTTLPR* and suicide risk [60,61,62,63,64].	No specific epigenetic studies mentioned.
BDNF	Association between Val66Met polymorphism in the *BDNF* gene and suicide in various psychiatric diagnoses. Mixed results in meta-analyses and replication studies [33,65,66,67,68,69,70,71,72,73].	Hypermethylation of the *BDNF* promoter region associated with reduced BDNF production in suicidal patients. Consistent findings in postmortem studies, showing significant hypermethylation in the brains of suicide victims [74,75,76,77,78].
HPA axis	Genetic polymorphisms: *NR3C1* (glucocorticoid receptor), *FKBP5*, and *CRHBP* genes increase suicide risk. Non-suppression of cortisol in DST is associated with higher suicide risk [79,80,81]. GABA: Polymorphisms in the *GABA* receptor subunit genes are linked to variations in HPA axis reactivity and increased suicide risk. GABA inhibits the HPA axis and locus coeruleus–norepinephrine system, affecting stress response [80,82,83].	Methylation of *NR3C1*, *SKA2*, and *CRH* receptor genes related to suicide risk. Childhood trauma linked to hypermethylation of HPA axis genes, leading to altered stress responses and increased susceptibility to suicide [79,84,85,86,87].
Second messengers	PKC and PKA: Decreased activity and expression of PKC and PKA in suicide victims. Significant decrease in Bmax of [3H] PDBu binding in the PFC of teenage suicide victims [88,89].	No specific epigenetic studies mentioned.
Inflammatory markers	Cytokines: Elevated IL-6 and IL-1B levels, *TNF-alpha* polymorphisms (rs1800629 and rs1799724) associated with suicide. Decreased IL-8 levels were found in suicidal patients [90,91,92,93].	miRNAs and methylation: miR-19a-3p regulation of TNF-alpha expression, hypermethylation of cytokine genes. Epigenetic changes linked to inflammation and suicide, with specific miRNAs and methylation patterns affecting cytokine production [94].

N/A: non-applicable.

## Data Availability

No new data were created or analyzed in this study. Data sharing is not applicable to this article.
